# A Clinical Impact Study of Myocardial Perfusion Imaging Comparing Three Different Cardiac Software Packages at 1-Year Follow-Up

**DOI:** 10.1055/s-0046-1819612

**Published:** 2026-03-18

**Authors:** Amal Paul, Ashwini Kalshetty, Sandip Basu

**Affiliations:** 1 Radiation Medicine Centre, Bhabha Atomic Research Centre, Tata Memorial Hospital Annexe, Mumbai, Maharashtra, India; 2 Homi Bhabha National Institute, Mumbai, Maharashtra, India

**Keywords:** myocardial perfusion imaging, quantification, receiver operating characteristic, cardiovascular disease, major adverse cardiac events

## Abstract

**Purpose:**

Myocardial perfusion imaging (MPI) aids in evaluating the left ventricular function and therefore helps in risk stratification and prognosis. The purpose of this study was to evaluate the clinical impact of three cardiac software packages (Emory Cardiac Toolbox, Cardiogam, and Myovation Evolution) at 1-year follow-up.

**Methods:**

This prospective investigation involved 370 patients who underwent myocardial perfusion scintigraphy. Participants were stratified into three groups based on left ventricular ejection fraction determined by two-dimensional echocardiography. Among these, 294 patients were available for follow-up at 1 year. Semiquantitative parameters (summed rest score [SRS], summed stress score [SSS], summed difference score [SDS]) and quantitative parameters (defect extent [DE], transient ischemic dilatation [TID], ejection fraction [EF], end-diastolic volume [EDV], end-systolic volume [ESV], and stroke volume [SV]) derived from each software platform (ECTb, Cgam, and ME) were used to evaluate their diagnostic performance in predicting major adverse cardiac events (MACE) using receiver operating characteristic curve analysis in patients during 1-year follow-up.

**Results:**

Among the 294 participants available for follow-up, 6% ( *n* = 17) developed MACE at 1 year and 88% ( *n* = 15) belonged to the high-risk group. Follow-up analysis indicated that all three software packages could predict 1-year cardiac events with varying cut-off values, sensitivities, and specificities. Cut-off values for SRS (ECTb) was ≥4 (area under the curve [AUC], 0.82), while for Cgam it was ≥11 (AUC 0.8), DE (REST) (ECTb) was ≥6% (AUC 0.82), and Cgam value ≥4.18% (AUC 0.81), EF (REST) was ≤44% (AUC 0.82), ≤68% (AUC 0.84) and ≤35% (AUC 0.83) for ECTb, Cgam, and ME, respectively, EDV (REST) was ≥137 mL for both ECTb (AUC 0.83) and Cgam (AUC 0.85) while for ME it was ≥116 mL (AUC 0.8), ESV (REST) was ≥91 mL (AUC 0.83), ≥55 mL (AUC 0.86) and ≥78 mL (AUC 0.82) for ECTb, Cgam, and ME, respectively.

**Conclusion:**

Majority of the MACE occurred in the group with high-risk characteristics. All three software packages demonstrated the ability to predict 1-year cardiac events, albeit with varying parameter cut-off values, sensitivities, and specificities.

## Introduction

Cardiovascular diseases (CVDs)—including ischemic heart disease (IHD), stroke, heart failure, peripheral and aortic artery disease, arrhythmias, and valvular disorders—remain the leading global cause of mortality and disability. In 2022, CVD accounted for an estimated 19.8 million deaths, 396 million years of life lost, and 44.9 million years lived with disability, with IHD showing the highest global age-standardized disability-adjusted life years at 2,275.9 per 100,000. 1 Myocardial perfusion imaging (MPI) is a cornerstone noninvasive tool for diagnosing and managing CVD, supported by advances in single-photon emission computed tomography (SPECT) technology, radiopharmaceuticals, and image-processing software. 2 MPI demonstrates strong diagnostic concordance with two-dimensional echocardiography (2D ECHO), 3 4 CT angiography, 5 6 7 and cardiac magnetic resonance imaging. 8 9 10 11 Several commercially available software packages support clinical decision-making in nuclear cardiology by enabling automated analysis of MPI using a reference normal database. 12 13 14 Based on the 17-segment model of the left ventricle, these software packages assign perfusion severity scores on a standardized 5-point scale. 15 MPI analysis provides semiquantitative parameters like summed rest score (SRS), summed stress score (SSS), summed difference score (SDS), defect extent (DE), transient ischemic dilatation, and quantitative parameters like ejection fraction (EF), end diastolic volume (EDV), end systolic volume (ESV), and stroke volume (SV) for the evaluation of left ventricular function. 16 Previously, we investigated the comparison and correlation of three software packages—ECTb, ME, and Cgam—with respect to 2D ECHO. The technological comparison study of the parameters obtained through the three different cardiac software packages will be reported separately. In this subset study of the previously mentioned technical comparison study, we evaluated the clinical impact of each software (ECTb, ME, and Cgam) during a 1-year follow-up in the prediction of major adverse cardiac events (MACE).

## Materials and Methods

This prospective study was performed over 18 months (June 2022–December 2023) following Institutional Ethics Committee approval and included 370 patients referred for MPI. Participants were grouped as: group I, low-risk individuals with preserved EF (>50%) and no prior cardiac disease; group II, patients with known IHD and preserved EF (>50%); and group III, high-risk patients with reduced EF (<50%) on 2D ECHO. All subjects underwent a standard 2-day rest–stress MPI protocol in accordance with American Society of Nuclear Cardiology guidelines, 17 using 99mTc-sestamibi (10–20 mCi) with treadmill (Bruce protocol), adenosine stress, or rest-only imaging when stress testing was not feasible. Imaging was performed on a GE Discovery 670 DR SPECT system with LEHR collimators. Rest images were acquired 60 minutes after injection and stress images 15 to 45 minutes post-tracer. Data were collected over a 180° noncircular orbit (64 × 64 matrix, zoom 1.3) with ECG gating when appropriate, supplemented by low-dose CT for attenuation correction. Reconstruction on the Xeleris 4DR workstation included corrections for nonuniformity, decay, and motion, followed by filtered back projection (Butterworth filter) and generation of short axis, vertical long axis, and horizontal long axis views. Semiquantitative and quantitative analysis—covering perfusion defects, image quality, and attenuation-corrected parameters—was performed using Emory Cardiac Toolbox, Cardiogam, and Myovation Evolution according to each software's analytical features. The parameters available for analysis are tabulated in Table 1 .

**Table 1:** Parameters available in ECTb, Cgam, and Myovation Evolution software

Emory Cardiac Toolbox	Cardiogam	Myovation Evolution
Perfusion parameters		
Polar maps (17 segments) show segmental scores (numerical), the scoring system is a 5-point scale: 0: Normal 1: Equivocal 2: Moderate reduction in perfusion 3: Severe reduction in perfusion 4: Absent perfusion	Polar maps (17 segments) show segmental scores (numerical), the scoring system is a 5-point scale: 0: Normal 1: Indeterminate 2: Abnormal 3: Severely abnormal 4: No perfusion	Polar maps (17 segments) show segmental pixel count (Max count 100).
Summed rest score	Summed rest score	
Summed stress score	Summed stress score	
Summed difference score	Summed difference score	
Defect extent	Extent (%)	
Defect severity	Severity	
Reversibility		
Transient ischemic dilatation		Transient ischemic dilatation
Estimated myocardial mass (gated)		
Estimated myocardial mass (ungated)		
Stress total severity score		
Rest total severity score		
Reversibility total severity score		
Probability of survival		
Functional parameters		
Ejection fraction	Ejection fraction	Ejection fraction
End diastolic volume	End diastolic volume	End diastolic volume
End systolic volume	End systolic volume	End systolic volume
Stroke volume		Stroke volume
Peak filling rate		Perfusion volume
Time to peak filling rate		
Peak filling rate index		
Heart rate		
Wall thickening	Wall thickening	
Wall motion	Wall motion	

### Follow-Up

Follow-up assessments were conducted at 3, 6, and 12 months during the 1-year follow-up period in the cardiology clinic with review of medical records to evaluate the following outcomes: post-MPI intervention (medical or surgical management), requirement of further investigations prior to management, symptomatic response from major baseline complaints of chest pain and dyspnea, and development of MACE. The MACE as defined in this study includes myocardial infarction (MI), heart failure, and cardiac event–related deaths during the 1-year follow-up period. 18 We evaluated the diagnostic performance of software-derived parameters at baseline MPI in the prediction of cardiac events at 1 year. Out of a total of 370 patients included in the study, 294 (79.5%) patients were available for follow-up.

### Statistical Analysis

Receiver operating characteristic (ROC) analyses were used to determine optimal cut-off values for these parameters. The nonparametric ROC curve, without covariates, was used to evaluate the semiquantitative and quantitative parameters derived from each software to predict MACE during the 1-year follow-up period. These parameters were treated as classification variables, while the binary cardiac event (Yes/No) served as the reference variable. An AUC of 0.8 or higher was considered indicative of good discriminative ability. The optimal cutoff for each semiquantitative and quantitative parameter was determined using the maximum Youden index (Sensitivity + Specificity − 1).

## Observation and Results

A total of 370 patients (mean age 58.8 years, range 14–86 years) who met the inclusion criteria were enrolled, and their clinical characteristics are summarized in Table 2 . In a separate study, we had investigated the comparative evaluation of the parameters derived from these three software packages and had shown good correlation, although few inter-software variations were present.

**Table 2:** Patient demography with clinical characteristics

Total cases	370
Male patients	225
Female patients	145
Chest pain	166
DOE	170
HTN	215
DM	173
Dyslipidemia	152
IHD	161
PTCA	52
CABG	14
Physical stress MPI	113
Pharmacological stress MPI	133
Rest only	124
Group I (low likelihood group)	129
Group II (K/c/o IHD with preserved EF > 50%)	120
Group III (EF < 50%)	121
Follow-up cases	294

Abbreviations: CABG, coronary artery bypass graft; DM, diabetes mellitus; DOE, dyspnea on exertion; HTN, hypertension; IHD, ischemic heart disease; MPI, myocardial perfusion imaging; PTCA, percutaneous transluminal coronary angioplasty.

### Follow-Up

**Fig. 1:**
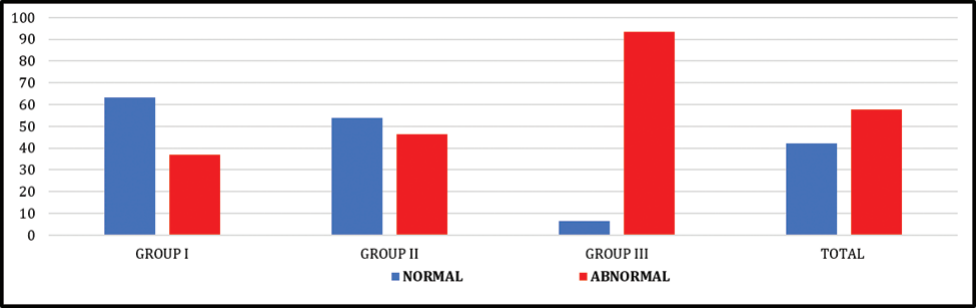
Percentage group-wise distribution of perfusion abnormality among follow-up cases. At follow-up, group I had *n* = 109, group II, *n* = 91, and group III, *n* = 94 patients. Total, *N* = 294 patients.

Out of a total of 370 patients included in the study, 294 (79.5%) patients were available for follow-up. Of these, 37.1% ( *n* = 109) were in group I, 30.9% ( *n* = 91) in group II, and 32% ( *n* = 94) in group III. Analysis of perfusion status showed that 57.8% ( *n* = 170) had abnormal perfusion, with the highest rate observed in group III at 93.6% ( *n* = 88). The distribution of perfusion abnormalities across groups is depicted in Fig. 1 .

Among the total of 294 individuals, 24.5% patients underwent additional diagnostic procedures prior to intervention, and the majority of the patients belonged to group III. In addition, 19 among the 32 patients in group III underwent coronary angiography as an additional investigation prior to intervention. The findings are illustrated in Table 3 .

**Table 3:** Group-wise distribution of follow-up cases requiring further investigation

Further investigation				
Group	*N*	ECG	2D ECHO	CAG
I: 109 (37.1%)	19 (17.4%)	1	2	16
II: 91 (30.9%)	21 (23.1%)	1	3	17
III: 94 (32%)	32 (34%)	5	8	19
Total *n* = 294	72 (24.5%)	7	13	52

Abbreviations: CAG, coronary angiography; ECG, electrocardiography; ECHO, echocardiography.

**Fig. 2:**
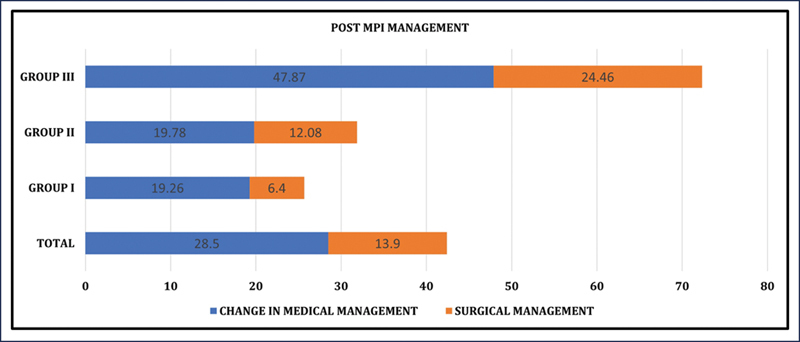
Percentage total and group-wise distribution undergoing change in intervention post-MPI study. MPI, myocardial perfusion imaging.

The evaluation of follow-up management after the MPI study, as shown in Fig. 2 , showed that 28.5% ( *n* = 84) of cases underwent changes in medical management, while 13.9% ( *n* = 41) underwent surgical interventions, like percutaneous transluminal coronary angioplasty (PTCA). Group III accounted for most management modifications, with approximately 48% ( *n* = 45) receiving adjusted medical treatment and approximately 24% ( *n* = 23) undergoing surgical procedures. Medical adjustments primarily involved adding cardiac remodeling–protective drugs, including angiotensin-converting enzyme inhibitors, angiotensin receptor blockers, and angiotensin receptor neprilysin inhibitors (e.g., sacubitril/valsartan). Among surgically treated group III patients, 14 underwent PTCA and 8 underwent coronary artery bypass graft. Symptomatic improvement was observed in 78% ( *n* = 84) of patients with medical management changes and 85% ( *n* = 35) of those who had surgical treatment. These findings are summarized in Table 4 .

**Table 4:** Total and group-wise distribution of patients showing improvement in baseline symptoms post-MPI intervention

	Improvement in baseline symptoms post-MPI intervention	
	Intervention		
Group	Change in medical management	Surgical management
I	22/26 (84.6%)	7/7 (100%)
II	21/24 (87.5%)	9/10 (90%)
III	41/57 (72%)	19/24 (79.2%)
Total	84/107 (78.5%)	35/41 85.4%

Abbreviation: MPI, myocardial perfusion imaging.

### Major Adverse Cardiac Events at Follow-Up (1 Year)

**Fig. 3:**
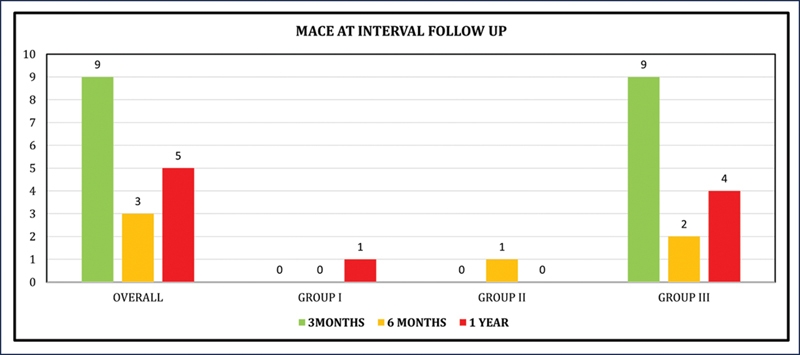
Overall and group-wise distribution of MACE at the interval and 1-year follow-up period. MACE, major adverse cardiac event.

Out of the total 294 individuals, 17 patients (6%) developed MACE at the 1-year follow-up period and the distribution of events among the various groups is tabulated in Table 5 . It was observed that approximately 88% ( *n* = 15) of patients developing MACE belonged to group III. Further subclassification of MACE at 3 months, 6 months, and 1 year is illustrated in a bar chart in Fig. 3 . It shows that the majority of cardiac events, 53% ( *n* = 9), developed at the initial 3 months of follow-up, and all these cases belonged to group III. Overall, 9 out of the 17 patients expired due to cardiac events, 5 patients developed heart failure, and 3 patients had acute MI.

**Table 5:** Group-wise distribution of follow-up cases according to MACE at 1-year follow-up period

Group	Total follow-up			Groupwise	
I: 109 (37.1%)	1	1/294	0.34%	1/109	0.9%
II: 91 (30.9%)	1	1/294	0.34%	1/91	1.1%
III: 94 (32%)	15	15/294	5%	15/94	16%
Total, *n* = 294	17	17/294	6%		

Abbreviation: MACE, major adverse cardiac events.

### Individual Software Analysis with Follow-Up Data

**Fig. 4:**
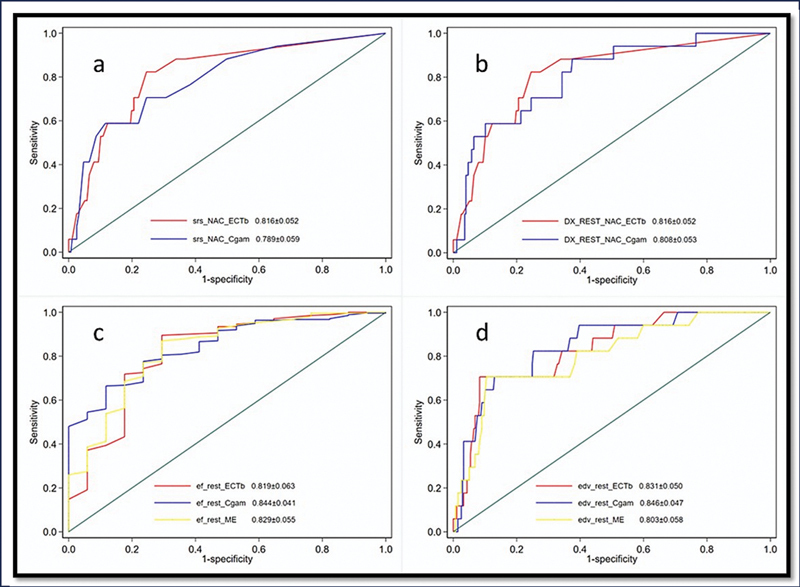
**(a–e)** ROC curve to predict MACE at 1-year period using (a) SRS (ECTb vs. Cgam) ( *p* < 0.001), (b) DEFECT EXTENT (REST) (ECTb vs. Cgam) ( *p* < 0.001), (c) EF at REST (ECTb vs. Cgam vs. ME) ( *p* < 0.001), and (d) EDV at REST (ECTb vs. Cgam vs. ME) ( *p* < 0.003). MACE, major adverse cardiac event; ROC, receiver operating characteristic; SRS, summed rest score.

ROC analysis was performed for all semiquantitative, quantitative, and functional parameters from each software to determine their ability to predict 1-year MACE. The significant parameters ( *p* < 0.05) with their cut-offs, accuracy, sensitivity, specificity, and AUC are summarized in Table 6 . For ECTb, an SRS ≥4 predicted MACE with an AUC of 82%, 82% sensitivity, and 76% specificity; for Cgam, an SRS ≥11 yielded an AUC of 79%, 59% sensitivity, and 89% specificity. DE at rest was also predictive, with cut-offs of ≥6% (ECTb) and ≥4.18% (Cgam), giving AUCs of 82 and 81%, sensitivities of 82 and 88%, and specificities of 76 and 63%, respectively. Resting EF cut-offs were ≤44% (ECTb), ≤68% (Cgam), and ≤35% (ME), with corresponding AUCs of 82, 84, and 83%; sensitivities of 90, 66, and 87%; and specificities of 71, 88, and 71%. The EDV threshold was ≥137 mL for both ECTb and Cgam (sensitivity 71%, specificity 92 and 87%, respectively), while ME showed a cut-off of ≥116 mL (sensitivity 70.6%, specificity 89.5%). Resting ESV cut-offs were ≥91 mL (ECTb), ≥55 mL (Cgam), and ≥78 mL (ME), with AUCs of 84, 86, and 82%, sensitivities of 71, 71, and 76%, and specificities of 91, 83, and 89%, respectively. Additionally, Cgam SV ≤69 mL showed an AUC of 65.1%, 52.9% sensitivity, and 76.5% specificity. Overall, all three software packages demonstrated predictive value using different parameter cut-offs, though ECTb generally showed slightly better sensitivity and specificity across most metrics. ROC plots for all parameters are shown in Fig. 4 **.**

**Table 6:** Analysis of parameters for predicting MACE at 1-year period significance ( *p* ;< 0.05)

Sr._no.	Predictor	Optimal cutoff	Sensitivity	Specificity	Accuracy	AUC	*p* -Value
1	SRS ECTb	(≥ 4)	0.8235	0.7545	0.7585	0.816	<0.001
2	SRS Cgam	(≥ 11)	0.5882	0.8845	0.8673	0.790	<0.001
3	DE (REST) ECTb	(≥ 6)	0.8235	0.7545	0.7585	0.816	<0.001
4	DE (REST) Cgam	(≥ 4.18)	0.8824	0.6245	0.6395	0.808	<0.001
5	EF (REST) ECTb	(≤ 44)	0.8953	0.7059	0.8844	0.819	<0.001
6	EF (REST) Cgam	(≤ 68)	0.6643	0.8824	0.6769	0.844	<0.001
7	EF (REST) ME	(≤ 35)	0.87	0.7059	0.8605	0.829	<0.001
8	EDV REST ECTb	(≥ 137)	0.7059	0.917	0.9048	0.831	<0.001
9	EDV REST Cgam	(≥ 137)	0.7059	0.87	0.8605	0.846	<0.001
10	EDV REST ME	(≥ 116)	0.7059	0.8953	0.8844	0.803	<0.001
11	ESV REST ECTb	(≥ 91)	0.7059	0.9061	0.8946	0.835	<0.001
12	ESV REST Cgam	(≥ 55)	0.7647	0.8339	0.8299	0.864	<0.001
13	ESV REST ME	(≥ 78)	0.7059	0.8917	0.881	0.823	<0.001
14	SV REST Cgam	(≤ 69)	0.5294	0.7653	0.7517	0.651	0.036
15	SV REST ECTb	(≤ 24)	0.9819	0.2353	0.9388	0.569	0.342
16	SV REST ME	(≤ 34)	0.6137	0.5882	0.6122	0.585	0.242

Abbreviations: AUC, area under the curve; DE, defect extent; EDV, end-diastolic volume; EF, ejection fraction; ESV, end-systolic volume; SRS, summed rest score; SV, stroke volume.

## Discussion

In the present study, we evaluated the clinical impact of MPI using three software packages—ECTb, ME, and Cgam—during a 1-year follow-up. Our study is one of the first studies using three cardiac software-derived semiquantitative and quantitative parameters to derive a cut-off to predict the occurrence of MACE at 1-year follow-up.

### Follow-Up

Among the 294 patients followed (79.5% of the cohort), 6% ( *n* = 17) experienced MACE at 1 year, with most events occurring in group III and 53% arising within the first 3 months. Prior studies similarly highlight the prognostic value of MPI. Zotou et al 19 reported a 7.1% 1-year MACE rate—including 4.6% adverse events and 2.6% mortality—in a high-risk cohort, whereas our lower proportion of high-risk patients (only 37% low-likelihood individuals) may explain the reduced event rate. Shaw and Iskandrian 20 found annual cardiac death or nonfatal MI rates of 5.9% in high-risk SPECT groups versus 0.6% in low-risk patients. Nakazato et al 21 observed rising annual event rates with increasing D-SPECT perfusion defect size, from 1.9% (small defects) to 5.3% (large defects). Similarly, Engbers et al 22 reported event rates of 0.6% for normal scans, 2.8% for small defects, and 4.3% for moderate to large defects.

Patients with abnormal MPI findings underwent either medical or surgical management, with most showing improvement in baseline symptoms. Surgical intervention resulted in greater symptomatic improvement than medical therapy (85 vs. 79%), particularly in group I (100 vs. 85%) and group III (79 vs. 72%). Similar findings were reported in the COURAGE trial, 23 where 70% of patients receiving percutaneous coronary intervention plus optimal medical therapy (OMT) and 63% receiving OMT alone were angina-free at 6 to 18 months. The BARDOT trial 24 also demonstrated improved survival and lower rates of MI and repeat revascularization in patients with abnormal MPI who underwent revascularization compared with those managed medically or not revascularized. The differences observed in our study likely reflect variations in patient populations across studies.

Analysis of statistically significant ( *p* < 0.05) software parameters for predicting 1-year cardiac events showed an SRS cut-off of ≥4 for ECTb and ≥11 for Cgam, with sensitivity and specificity as reported in Table 6 . No prior studies reported SRS cut-offs for ECTb or Cgam; only Angelidis et al 25 evaluated Myovation-derived SRS, finding a cut-off of ≥4.5 predicted events with AUC 0.65, sensitivity 69.6%, and specificity 53.3%. Similarly, Gimelli et al 26 reported SRS ≥4 identified patients at risk, with sensitivity 48% and specificity 70%. Our ECTb cut-off of ≥4 aligns with these findings but showed higher sensitivity, supporting the idea that SRS reflects normalized uptake independent of myocardial contractility, hemodynamics, or medical therapy. For DE at rest, cut-offs were ≥6% (ECTb) and ≥4.18% (Cgam). Garcia et al 27 suggested a threshold of 3 to 5% for abnormal scans, while Berman et al 28 found 5% hypoperfused myocardium at stress optimally separated low (∼1%/y) versus higher risk patients. For functional parameters, EF cut-offs were ≤44% (ECTb), ≤68% (Cgam), and ≤35% (ME), with higher sensitivity for ECTb and ME. EDV cut-offs were ≥137 mL for ECTb and Cgam and ≥116 mL for ME, with similar sensitivity but better specificity for ECTb and ME. ESV cut-offs were ≥91 mL (ECTb), ≥55 mL (Cgam), and ≥78 mL (ME), with comparable sensitivities and higher specificity for ECTb and ME. Ababneh et al 29 reported normal-gated SPECT ranges: EF lower limits 50% for women, 43% for men, EDV upper limits 91 mL (women), 119 mL (men), and ESV 40 mL (women), 55 mL (men). Sharir et al 30 showed post-stress left ventricular ejection fraction (LVEF) >45% and ESV ≤70 mL predicted low annual cardiac death (1.7%) versus EF <45% and ESV >70 mL (7.9%, *p* < 0.02). Differences in mean EF, EDV, and ESV in our study may reflect variations in study populations, follow-up periods, MPI acquisition, software, and reconstruction protocols. Notably, previous studies reported cut-offs for abnormality rather than for predicting cardiac events, which may explain the variations observed in our cohort. It is commonly known that the semiquantitative and quantitative parameters vary across different software packages, although they show similar diagnostic performance. Guner et al reported comparable accuracy for detecting coronary artery disease across ECTb, 4D-MSPECT, and Quantitative Perfusion SPECT, despite notable variation in their numerical outputs. 31 Similarly, Wolak et al found differences in automation, diagnostic performance, and perfusion quantification, with only moderate to strong correlations among SSS values. 32 Svensson et al also observed inter-software disagreement—particularly for SRS and SSS, and to a lesser extent SDS—when comparing Cedars QPS, ECTb, and 4D-MSPECT. 33 In our previous study validating the semiquantitative and functional parameters derived from three software packages, ECTb, Cgam, and ME, it was also shown that although there was good correlation between the parameters, notable inter-software differences existed between them. For predicting 1-year MACE, ECTb demonstrated superior sensitivity and specificity compared with ME and Cgam, with values comparable to previous studies. Study limitations included variability in acquisition timing, non-protocol-adherent automated reconstruction, and use of different software with varying normal databases and definitions of abnormal pixels. Limited stress testing and a high proportion of rest-only studies (often due to low EF or large perfusion defects) restricted attenuation correction. Follow-up was affected by a 20.5% dropout rate and a predominance of low-risk patients, limiting generalizability. Additionally, detailed post-management data were missing for many patients, and uncertainty regarding the cause of death necessitated using all-cause mortality as the primary clinical outcome.

## Conclusion

Follow-up analysis demonstrated that each software was able to predict 1-year cardiac events using distinct cut-off values, with variable sensitivity and specificity. Overall, ECTb showed superior predictive performance for MACE at 1 year, consistent with previously reported findings. This study underscores the value of SPECT MPI in prognostication and risk stratification, highlighting the clinical utility of multiple nuclear cardiology software.
